# *S*‐Nitrosated alpha‐1‐acid glycoprotein exhibits antibacterial activity against multidrug‐resistant bacteria strains and synergistically enhances the effect of antibiotics

**DOI:** 10.1096/fba.1018

**Published:** 2019-02-04

**Authors:** Yu Ishima, Kaori Watanabe, Victor T. G. Chuang, Iyo Takeda, Teruo Kuroda, Wakano Ogawa, Hiroshi Watanabe, Yasunori Iwao, Tatsuhiro Ishida, Masaki Otagiri, Toru Maruyama

**Affiliations:** ^1^ Department of Pharmacokinetics and Biopharmaceutics Institute of Biomedical Sciences, Tokushima University Tokushima Japan; ^2^ Department of Biopharmaceutics, Graduate School of Pharmaceutical Sciences Kumamoto University Kumamoto Japan; ^3^ School of Pharmacy Monash University Malaysia Subang Jaya, Selangor Malaysia; ^4^ Department of Microbiology Institute of Biomedical & Health Sciences, Hiroshima University Hiroshima Japan; ^5^ Department of Microbiology and Biochemistry Daiichi University of Pharmacy Fukuoka Japan; ^6^ Department of Pharmaceutical Engineering, Graduate School of Pharmaceutical Sciences University of Shizuoka Shizuoka Japan; ^7^ Faculty of Pharmaceutical Sciences Sojo University Kumamoto Japan

**Keywords:** alpha‐1‐acid glycoprotein, antimicrobial effect, multidrug resistance, nitric oxide, S‐nitrosation

## Abstract

Alpha‐1‐acid glycoprotein (AGP) is a major acute‐phase protein. Biosynthesis of AGP increases markedly during inflammation and infection, similar to nitric oxide (NO) biosynthesis. AGP variant A (AGP) contains a reduced cysteine (Cys149). Previously, we reported that *S*‐nitrosated AGP (SNO‐AGP) synthesized by reaction with a NO donor, possessed very strong broad‐spectrum antimicrobial activity (IC_50 _= 10^−9^‐10^−6^ M). In this study, using a cecal ligation and puncture animal model, we confirmed that AGP can be endogenously *S*‐nitrosated during infection. Furthermore, we examined the antibacterial property of SNO‐AGP against multidrug‐resistant *Klebsiella pneumoniae* and *Pseudomonas aeruginosa* to investigate the involvement of SNO‐AGP in the host defense system. Our results showed that SNO‐AGP could inhibit multidrug efflux pump, AcrAB‐TolC, a major contributor to bacterial multidrug resistance. In addition, SNO‐AGP decreased biofilm formation and ATP level in bacteria, indicating that SNO‐AGP can revert drug resistance. It was also noteworthy that SNO‐AGP showed synergistic effects with the existing antibiotics (oxacillin, imipenem, norfloxacin, erythromycin, and tetracycline). In conclusion, SNO‐AGP participated in the host defense system and has potential as a novel agent for single or combination antimicrobial therapy.

**Abbreviations**
AGP: alpha‐1‐acid glycoprotein
CLP: cecal ligation and puncture
EPS: extracellular polymeric substance
SNO‐AGP: *S*‐nitrosated AGP

## 1 INTRODUCTION

While antimicrobial resistance emerges rapidly, unfortunately new antimicrobial agent discovery progresses very slowly. Due to the uncontrolled large quantity use of antibiotics, infections by resistant bacteria, such as methicillin‐resistant *Staphylococcus aureus* (MRSA) and multidrug‐resistant *Streptococcus pneumoniae*, are increasing. On the other hand, *Klebsiella pneumoniae* is the causative bacterium for respiratory infection, urinary tract infection, liver/biliary tract infection, septicemia, meningitis, and peritonitis. The second‐ and third‐generation cephalosporin antibiotics, and new quinolone antibiotics have been used to treat infections caused by *K. pneumoniae*, but with suboptimal therapeutic effect. Lately, the presence of extended‐spectrum beta‐lactamase–producing *K. pneumoniae* 1, 2, 3 and carbapenemase‐producing *K. pneumoniae*–resistant to carbapenem antibiotics and beta‐lactam antibiotics have been reported.^4^, ^5^, ^6^

Furthermore, multidrug‐resistant *Pseudomonas aeruginosa* is an increasing concern for clinicians,^7^ owing to a broad resistance of the bacteria to not only beta‐lactam but also carbapenems, quinolones, aminoglycosides antibiotics.^6^, ^8^ Furthermore, this drug‐resistant *P. aeruginosa* readily infects patients with hematologic malignancy or other solid cancer, highly immunocompromised patients such as bone marrow or organ transplant patients, eventually leads to sepsis and pneumonia that become refractory with poor prognosis.^9^ In view of the above situations, development of new antibacterial agents as well as highly effective treatment strategy such as combination of synergistic antimicrobial agents is desirable.

Nitric oxide (NO) plays a crucial immunological role as a broad‐spectrum antimicrobial agent in various infections.^10^, ^11^, ^12^ However, NO is highly reactive with a short biological half‐life. Therefore, a NO carrier or NO‐generating agent needs to be developed for clinical application of NO as an antimicrobial drug. It is well known that *S*‐nitrosothiols, a stable NO reservoir, have a 10^2^‐ to 10^3^‐fold stronger antimicrobial activity than NO alone.^13^, ^14^ Hence, *S*‐nitrosothiols could be developed into a stable NO‐related antimicrobial drug. Interestingly, delamanid, a new antituberculosis medication, possesses the proposed mechanism of action involving intracellular NO release.^15^

Alpha‐1‐acid glycoprotein (AGP), an acute‐phase protein, is mainly produced in the liver. It is also produced by immune cells, such as monocytes and macrophages, primarily during inflammation, resulting in locally high AGP concentrations at inflammatory sites. In most individuals, AGP is a mixture of two main genetic variants, F1*S and A. While F1*S variants do not possess any free cysteine residue, variant A has one reduced cysteine residue at position 149 (Cys149).^16^ Although it is known that Cys149 of AGP is a binding site for copper, the biological roles of Cys149 are unclear. All these findings led us to hypothesize that AGP could acquire antibacterial activity through S‐nitrosation and may be a suitable NO carrier for use as an infectious disease therapeutic agent.

Our previous report demonstrated that (a) Cys149 of AGP could be exogenously *S*‐nitrosated by NO donor in vitro, (b) *S*‐nitrosated AGP (SNO‐AGP) strongly possessed a broad‐spectrum antimicrobial activity (IC_50_ = 10^−9^‐10^−6^ M) in vitro, and (c) exogenous SNO‐AGP exhibited superior antibacterial activity in vivo in cecal ligation and puncture (CLP) model mice and significantly improved their survival.^14^ However, it was unknown whether AGP could be endogenously *S*‐nitrosated in vivo.

In this study, we demonstrated that the concentration of AGP, as well as that of NO, increased remarkably during bacterial infection. In this context, we further attempted to detect the SNO‐AGP during bacterial infection in CLP model mice. Finally, we evaluated the functions of SNO‐AGP as antibacterial agent and chemical sensitizer against multidrug‐resistant *K. pneumoniae* and *P. aeruginosa*.

## 2 MATERIALS AND METHODS

### 2.1 Materials

Lyophilized AGP, methyl methanethiosulfonate (MMTS), dithiothreitol (DTT), and glutathione were purchased from Sigma‐Aldrich (St. Louis, MO). NaNO_3_ was obtained from Nacalai Tesque (Kyoto, Japan). Diethylenetriaminepentaacetic acid (DTPA) and *S*‐nitorsoglutathione (GSNO) were obtained from Dojindo Laboratories (Kumamoto, Japan). *N*‐[6‐(Biotinamido)hexyl]‐3′‐(2′‐pyridyldithio) propionamide (biotin‐HPDP) was obtained from Pierce (Rockford, IL). Other chemicals were of highest grade commercially available.

### 2.2 Preparation of SNO‐AGP

First, to reduce Cys149 of AGP, AGP was treated with DTT in potassium phosphate buffer (PPB) pH 7.4, for 5 minutes at 37°C, as reported previously.^14^ After removal of excess DTT by gel filtration (PD‐10 Desalting Columns; GE Healthcare Japan, Tokyo, Japan), DTT‐treated AGP (300 µM) was reacted with 3 mM GSNO in PPB containing 1 mM DTPA, pH 8.0, for 30 minutes at 37°C. Then, the excess GSNO was removed from SNO‐AGP solution by PD‐10 Desalting Columns, and was concentrated by ultrafiltration; it was stored at −80°C until use. The *S*‐nitroso moiety of SNO‐AGP was quantified by the HPLC method,^10^ and was found to be 0.32 ± 0.06 mol SNO/mol AGP.

### 2.3 Preparation of the CLP Model and detection of SNO‐AGP

Bacterial infection was induced by CLP; male ICR mice underwent CLP according to a previously reported method.^17^ This animal experiments were conducted in accordance with the ethical standards of the Institutional Animal Care and Use Committee of the University of Tokushima (T28‐39). Male ICR outbred mice greater than 5 weeks of age (25‐30 g). Blood samples (0.3 mL) from surviving mice were collected from the tail vein at 0, 3, 6, 9, 12, and 24 hours after the puncture. Nitrate (NO_3_^−^) levels of these serums were measured using an EiCOM ENO‐20 NOx analyzer (EiCOM, Kyoto, Japan). NaNO_3_ solutions were used to prepare a standard curve. The serum was collected from the tail vein at 12 by centrifugation (1500× *g*, 5 minutes, 4°C). Protein concentrations were adjusted to 0.5 mg/mL, followed by treatment with MMTS to block free SH, according to a previously reported method with minor modifications.^18^ To block free SH groups on the protein without affecting the disulfide bonds, 4 volumes of blocking buffer [225 mM HEPES (pH 7.7), 0.9 mM EDTA, 0.09 mM Neocuproine, 2.5% SDS, and 20 mM MMTS] were added. The resulting solutions were agitated for 20 minutes at 50°C. Proteins were then recovered by precipitation with acetone (final concentration, 50%), and the precipitates were resuspended in 0.1 mL of HENS buffer (protein concentration, 10 mg/mL). To this protein solution, 0.1 mL of biotin‐HPDP (4 mM) in *N,N*‐dimethylformamide and 0.1 mL of aqueous ascorbate (0 or 1 mM) with 100 µM CuCl were added, and the mixture was incubated for 1 hour at 25°C. Proteins were again recovered via acetone precipitation. To detect *S*‐nitrosated proteins labeled with HPDP‐biotin, western blot was carried out using peroxidase‐conjugated anti‐biotin antibodies.

### 2.4 Calculation of IC_50_ for antibiotics and *S*‐nitrosated proteins against multidrug‐resistant bacteria

Antibacterial activity against multidrug‐resistant gram‐negative bacteria *K. pneumoniae* MGH78578 and *P. aeruginosa* PAO 1 was evaluated according to a previously reported method with slight modification.^14^ Oxacillin, cefmetazole, imipenem, norfloxacin, erythromycin, kanamycin, tetracycline, chloramphenicol, SNO‐AGP, and *S*‐nitrosated human serum albumin (SNO‐HSA) were used as antimicrobial agents. For the cultivation, M9 supplemented with 0.1% yeast extract was used. In the above medium, *K. pneumoniae* MGH78578 or *P. aeruginosa* PAO 1 were prepared to OD_630_ = 0.050 ± 0.009 and used for the experiment. Each antimicrobial substance was added to the medium and reacted at 37°C for 9 hours. The turbidity (OD_630_) was measured, and the bacterial growth (%) was calculated by comparing with the control (PBS) group.

### 2.5 Combination effect of antibacterial agent and SNO‐AGP in multidrug‐resistant bacteria

A multidrug‐resistant strain of *K. pneumoniae* MGH78578 was grown in M9 medium and adjusted to OD_630_ = 0.051 ± 0.01. Each concentration of SNO‐AGP was added and grown in M9 medium at 37°C for 5 hours (OD_630_ = 0.2‐0.3). Thereafter, it was washed three times with M9 medium by centrifugation (10,000× *g*, 2 minutes) and used for the experiment. Four millimolar lactate was added to the pellets, and antimicrobial agents with ¼ × IC_50_ concentrations were also added. As antimicrobial agents, oxacillin, cefmetazole, imipenem, norfloxacin, erythromycin, tetracycline, chloramphenicol, and kanamycin were used. After incubation at 37°C for 6 hours, the turbidity (OD_630_) was measured, and the bacterial growth (%) was calculated by comparing with the control (PBS) group. For the determination of synergistic effect, the isobologram method was used.^19^

### 2.6 Effect of SNO‐AGP on biofilm formation

The biofilm was prepared using a 96‐well microplate (Cellstar 96 well cell culture plate, greiner bio‐one, Frickenhausen, Germany). *K. pneumoniae* was grown in M9 medium and adjusted to OD_630_ in each well of a 96‐well microplate. The biofilm formation was confirmed from 9 hours after culture. Therefore, SNO‐AGP was added to the culture supernatant, and the cells were statically cultured at 37°C for 9, 24, or 48 hours. After culturing, the medium was gently removed, and the remaining on the well and bottom was defined as a biofilm. After culturing, the biofilm was stained with 200 µL of 2% (w/v) crystal violet aqueous solution for 30 minutes. This method is a quantitative method utilizing the primary correlation between the adsorption amount of crystal violet dye and the dry weight of biofilm formed on the well and bottom.^19^ Then, the crystal violet solution was gently removed, sterilized water (250 µL) was added, pipetted 10 times, and the water was removed. This wash step was repeated twice. Immediately after washing, 200 µL of 95% ethanol was added and decolorized by allowing to stand at 25°C for 30 minutes. Ethanol (100 µL) in the supernatant was transferred to another 96‐well microplate and the biofilm was determined by measuring the absorbance at 570 nm.

### 2.7 Effect of SNO‐AGP on substrate accumulation of multidrug efflux pump

A multidrug‐resistant strain of *K. pneumoniae* MGH 78578 was grown in M9 medium and adjusted to OD_630_ = 0.050 ± 0.008. Each concentration of SNO‐AGP was added to the medium and grown in M9 medium at 37°C for 5 hours (OD_630_ = 0.2‐0.3). Thereafter, it was washed three times with M9 medium by centrifugation (10 000× *g*, 2 minutes) and used for the experiment. Four millimolar lactate and 100 μM carbonyl cyanide *m*‐chlorophenyl hydrazone (CCCP) as energy sources of multidrug efflux pump were added. Then, 1 mM norfloxacin, 20 μM EtBr, and/or 20 μM rhodamine 6G as substrates of multidrug efflux pump were added, incubated at 25°C for 15 min, and then wash three times by the medium (10 000× *g*, 2 minutes). The measurement was performed at an excitation wavelength of 485 nm and a emission wavelength of 535 nm. To identify types of multidrug efflux pump contributed to substrate accumulation, multidrug efflux pump AcrB‐deficient strains (*K. pneumoniae* SKY2/pSTV28) and AcrB‐overexpressed strains (*K. pneumoniae* SKY2/pKAC28M) were also used as AcrAB‐knockout and ‐introduced stains, respectively.^20^, ^21^

### 2.8 Measurement of ATP level in bacteria

The BacTiter‐Glo™ Microbial Cell Viability Assay (Promega, Madison, WI) was used according to the manufacturer's instructions. After reacting *K. pneumoniae* with SNO‐AGP at 37°C, an equal amount of reagent was added to the culture medium and incubated at 25°C for 5 minutes, then luminescence was measured using a multi‐microplate reader. The measurement was adjusted by the number of bacteria.

### 2.9 Detection of intracellular NO and reactive oxygen species (ROS) in bacteria

*K. pneumoniae* was grown to OD_630_ = 0.050 ± 0.008 in M9 medium, and each SNO‐AGP was added to the medium and reacted at 37°C for 7 hours. Thereafter, DAF‐FMDA (for NO) or CM‐H_2_DCFDA (for ROS) was added and reacted at 37°C for 1 hour, then the supernatant was removed by centrifugation (10 000× *g*, 2 minutes). The bacteria was resuspended in M9 medium, SPECTRA FLUOR XFluor 4 (TECAN) was used for monitoring at an excitation wavelength of 485 nm and a emission wavelength of 535 nm.

### 2.10 Dot‐blot analysis of nitrotyrosine on bacterial membrane

*K. pneumoniae* was treated with SNO‐AGP at 37°C for 30 minutes. Nitrative stress derived from AGP was assessed by dot‐blot analysis of a bacterial membrane. The SNO‐AGP‐treated bacteria was lysed using LIPA buffer (adjust to1 mg/mL), and the lysed bacteria (10 µL) was doted to a nitrocellulose membrane, and dried for 15 minutes at 60°C. For detection of nitrated protein, a mouse monoclonal 3‐nitrotyrosine antibody was used at a dilution of 1:1,000. Densitometric quantification of the dots was performed using ImageJ software.

### 2.11 Statistical Analysis

Data are shown as means ± SD for the indicated number of animals. Significant differences among each group were determined by means of the two‐tailed unpaired Student's *t* test. A probability *P* value of <0.05 was said to indicate statistical significance.

## 3 RESULTS

### 3.1 Formation of SNO‐AGP during CLP‐induced bacterial infection

**Figure 1:**
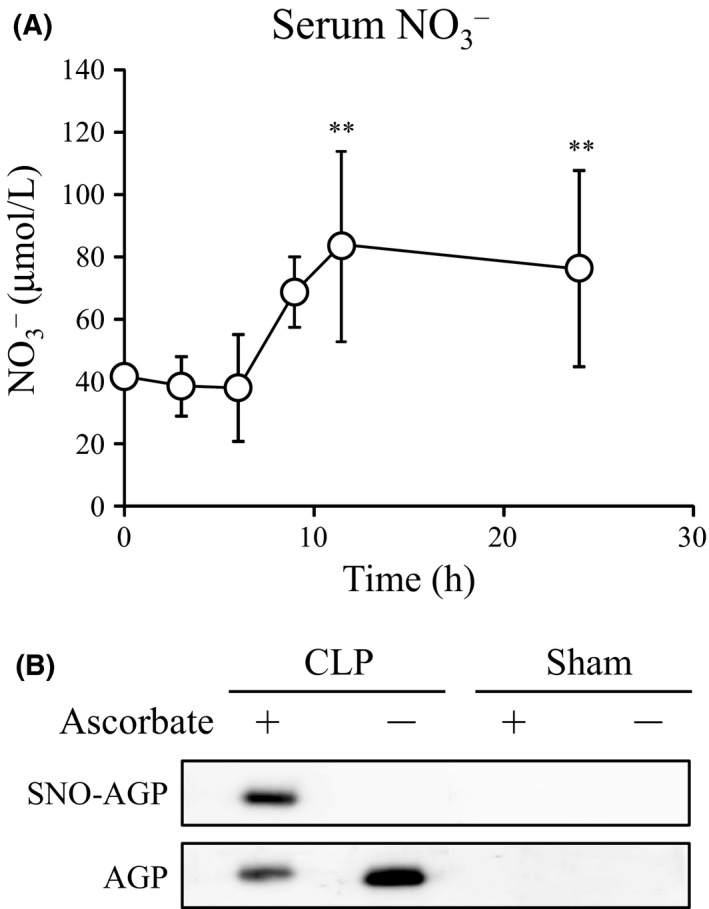
NO and SNO‐AGP production in mice CLP model. (A) Bacterial infection was induced by CLP, Blood samples from surviving mice were collected from the tail vein at 0, 3, 6, 9, 12, and 24 hours after the puncture. Nitrate (NO_3_^‐^) levels of these serums were measured using an EiCOM ENO‐20 NOx analyzer. Data are expressed as means ± SD (n = 5). **, *P *< 0.01 compared with at 0 hour after the puncture. (B) The serum collected from the tail vein at 12 hours after the puncture was collected by centrifugation. AGP and SNO‐AGP were detected using western blot and biotin switch assay, respectively. Ascorbate (‐) was used as negative control of biotin switch assay and this data shows the average of triplicate samples

During bacterial infection, inducible nitric oxide synthase (iNOS) and AGP were induced. We demonstrated that bacterial infection model by CLP actually increased the levels of nitrate and AGP in serum (Figure 1). We detected SNO‐AGP during CLP‐induced infection using a biotin switch assay, which is a detection method of *S*‐nitrosated proteins, suggesting that SNO‐AGP was endogenously induced during bacterial infection.

### 3.2 Antibacterial activity of SNO‐AGP against multidrug‐resistant bacteria

**Figure 2:**
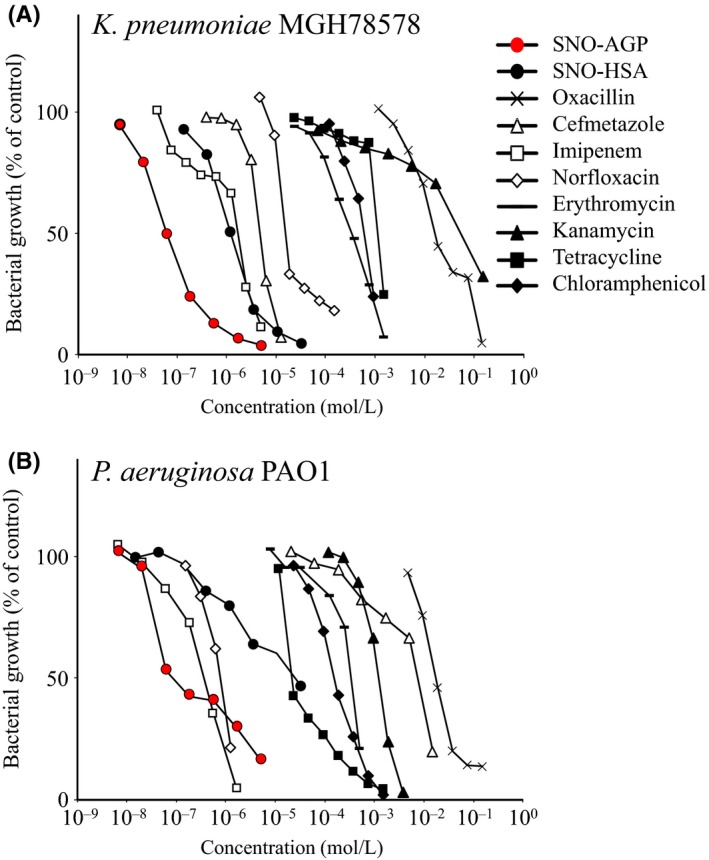
Antibacterial activity of *S*‐nitrosated proteins and antibiotics against (A) *Klebsiella pneumoniae* MGH78578 (B) *Pseudomonas aeruginosa* PAO1. *K. pneumoniae* MGH78578 or *P. aeruginosa* PAO 1 were prepared and used for the experiment. Each antimicrobial substance was added to the supernatant of the medium and reacted at 37°C for 9 hours. The turbidity (OD_630_) was measured, and the bacterial growth (%) was calculated by comparing with the control group. Data are expressed as means ± SD (n = 15)

We have showed that *S*‐nitrosated proteins possess antibacterial activity.^10^ Multidrug‐resistant bacteria *K. pneumoniae* 22 and *P. aeruginosa* 23, 24, 25 were used to compare the antibacterial activity of SNO‐AGP with existing antimicrobials (oxacillin, cefmetazole, imipenem, norfloxacin, erythromycin, kanamycin, tetracycline, and chloramphenicol). IC_50_ values for each antimicrobial agent against multidrug‐resistant bacteria were calculated. The IC_50_s of SNO‐AGP against *K. pneumoniae* and *P. aeruginosa* are 0.06 µM, and 1 µM, respectively (Figure 2 and Table 1). Our previous study demonstrated that AGP without NO addition did not affect bacterial growth.^14^ These data demonstrated that SNO‐AGP acts as an endogenous antibacterial agent in vivo.

**Table 1:** IC_50_ values for each antimicrobial agent against multidrug‐resistant bacteria

		IC_50_ (mM)	
Classification	Antibacterial agents	*K. pneumoniae*	*P. aeruginosa*
β‐Lactam	Oxacillin	15	15
	Cefmetazole	0.012	9
	Imipenem	0.002	0.0005
Quinolone	Norfloxacin	0.015	0.001
Aminoglycoside	Erythromycin	0.6	0.4
	Tetracycline	1.2	0.01
	Chloramphenicol	1.0	0.1
Macrolide	Kanamycin	100	0.7
RS‐NO	SNO‐AGP	0.00006	0.001
	SNO‐HSA	0.0013	0.025

### 3.3 Combined effect of various antimicrobial agents and SNO‐AGP against multidrug‐resistant bacteria

**Figure 3:**
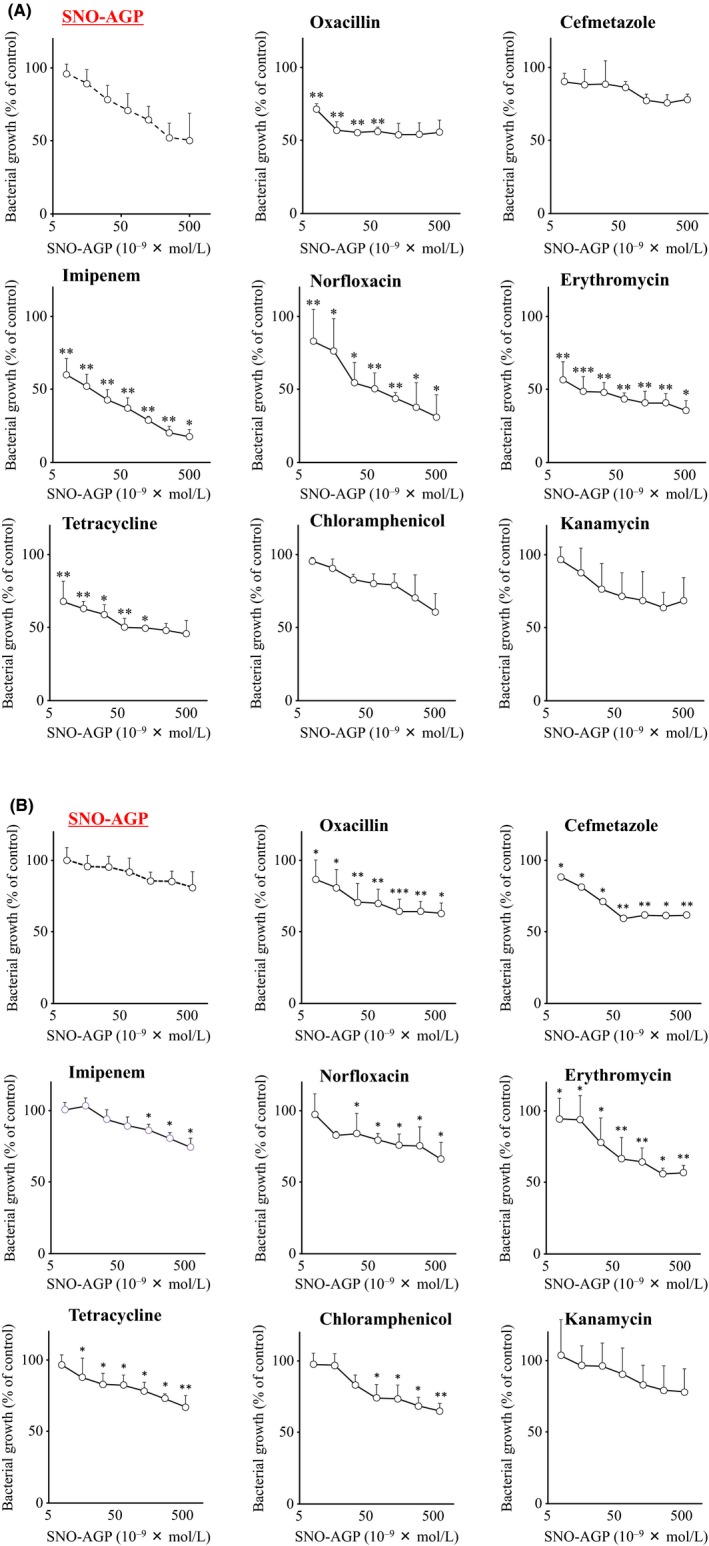
Growth inhibitory effect of antibacterial drugs combined with SNO‐AGP on multidrug‐resistant strains. Multidrug‐resistant strains of (A) *Klebsiella pneumoniae* MGH78578 or (B) *Pseudomonas aeruginosa* PAO1 were grown in M9 medium. Each concentration of SNO‐AGP was added to the medium supernatant and grown in M9 medium at 37°C for 5 hours (OD_630_ = 0.2‐0.3). Thereafter, it was washed three times with M9 medium and used for the experiment. Then, ¼ × IC_50_ concentrations of antimicrobial agents (oxacillin, cefmetazole, imipenem, norfloxacin, erythromycin, tetracycline, chloramphenicol, and kanamycin) were added and incubated at 25°C for 15 minutes. After incubation at 37°C for 6 hours, the turbidity (OD_630_) was measured and the bacterial growth (%) was calculated by comparing with the control group. Data are expressed as mean ± SD (n = 5). ***P < 0.001, **P < 0.01, *P < 0.05, compared with SNO‐AGP alone/drug (‐)

The combined effects of SNO‐AGP with various antimicrobial agents were evaluated using *K. pneumoniae* and *P. aeruginosa*. Each concentration of SNO‐AGP added to the medium was combined with or without an antimicrobial substance at a concentration of 1/4 of the IC_50_. The bacterial growth of *K. pneumoniae* and *P. aeruginosa* were significantly inhibited by SNO‐AGP in a concentration‐dependent manner. In addition, the antimicrobial activity of SNO‐AGP against *K. pneumoniae* was further inhibited by oxacillin, imipenem, norfloxacin, erythromycin, or tetracycline. On the other hand, the antimicrobial activity of SNO‐AGP against *P. aeruginosa* was also inhibited by oxacillin, cefmetazole, imipenem, norfloxacin, erythromycin, tetracycline, or chloramphenicol (Figure 3). Tables 2 and 3 showed combination index (CI) of the results calculated by the statistical analysis method isobologram method. If the value of CI = da/Da + db/Db is 1 or less, it is synergistic, 1 is additive, and if it is 1 or more, it is judged to be antagonistic (da, db; respective concentrations in combination with drugs a and b; Da, Db: concentrations of a and b alone necessary for showing the same effect as in combination). These data indicated that SNO‐AGP could synergistically enhance the antibacterial activity of a broad‐spectrum antimicrobial agent against these two multidrug‐resistant bacteria.

**Table 2:** IC_50_ values and combination index (CI) for each antimicrobial agent against *Klebsiella pneumoniae*

Antibacterial agents	IC_50_ (mM)	Drugs concn (mM)	CI^a^ in the presence of SNO‐AGP at concn (M) of:						
			0.00781	0.0156	0.0313	0.0625	0.125	0.25	0.5
Oxacillin	15	3.75	0.49	0.51	0.44	0.65	0.97	1.70	3.18
Cefmetazole	0.012	0.003	1.38	1.77	2.74	3.89	4.29	7.86	14.98
Imipenem	0.002	0.0005	0.48	0.44	0.38	0.38	0.28	0.27	0.30
Norfloxacin	0.015	0.00375	0.67	0.68	0.42	0.55	0.48	0.72	0.31
Erythromycin	0.6	0.15	0.79	0.57	0.58	0.55	0.63	0.43	0.40
Tetracycline	1.2	0.3	0.49	0.58	0.67	0.54	0.75	0.86	0.93
Chloramphenicol	1.0	0.25	2.45	2.60	2.34	3.04	5.00	4.13	3.79
Kanamycin	100	25	—	4.55	9.31	1.03	1.51	1.70	3.37

As antimicrobial agents, oxacillin, cefmetazole, imipenem, norfloxacin, erythromycin, tetracycline, chloramphenicol, and kanamycin were used.

a Combination index was calculated by the isobologram method.^19^

**Table 3:** IC_50_ values and combination index (CI) for each antimicrobial agent against *Pseudomonas aeruginosa*

Antibacterial agents	IC_50_ (mM)	Drugs concn (mM)	CI^a^ in the presence of SNO‐AGP at concn (M) of:						
			0.00781	0.0156	0.0313	0.0625	0.125	0.25	0.5
Oxacillin	15	3.75	0.73	0.27	0.12	0.14	0.15	0.21	0.25
Cefmetazole	9	2.25	0.67	0.57	0.43	0.28	0.30	0.35	0.45
Imipenem	0.0005	0.000125	1.18	2.09	1.81	0.84	1.04	1.19	1.17
Norfloxacin	0.001	0.00025	1.51	0.75	0.79	0.80	0.75	0.88	0.75
Erythromycin	0.4	0.10	0.90	0.98	0.64	0.36	0.38	0.35	0.43
Tetracycline	0.01	0.0025	0.67	0.34	0.38	0.48	0.47	0.32	0.35
Chloramphenicol	0.1	0.020	0.93	0.97	0.50	0.40	0.40	0.43	0.44
Kanamycin	0.7	0.175	1.45	1.95	2.46	2.40	1.05	1.07	1.66

As antimicrobial agents, oxacillin, cefmetazole, imipenem, norfloxacin, erythromycin, tetracycline, chloramphenicol, and kanamycin were used.

a Combination index was calculated by the isobologram method.^19^

### 3.4 Effect of SNO‐AGP on biofilm formation

**Figure 4:**
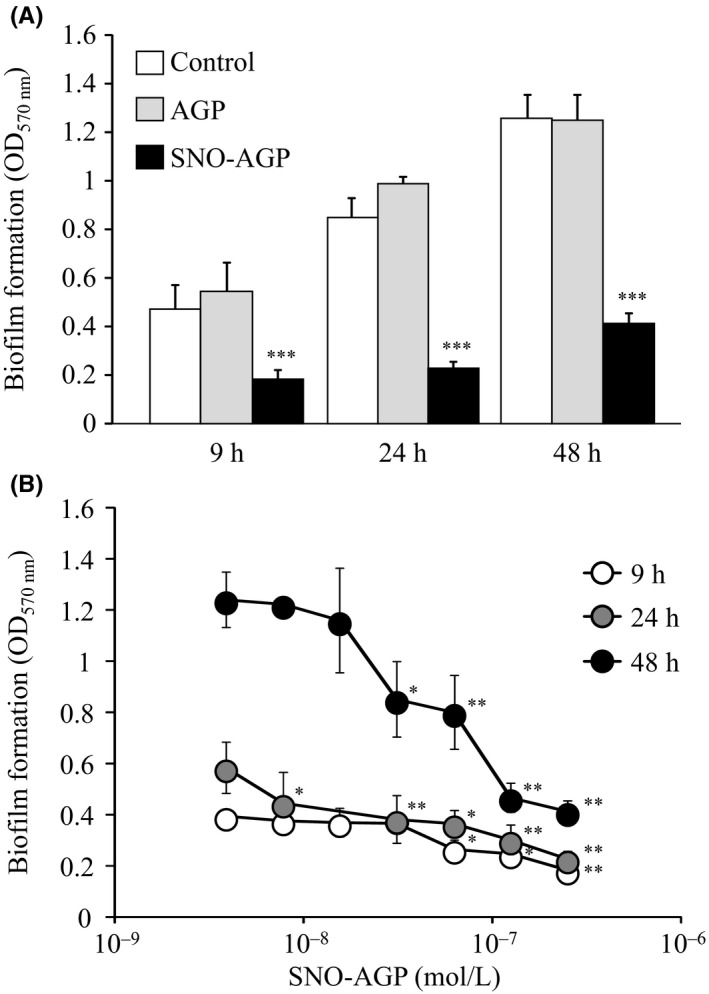
Quantification of biofilm formation of *Klebsiella pneumoniae* MGH78578 incubated with SNO‐AGP by crystal violet staining. *Pseudomonas pneumoniae* was grown in M9 medium and adjusted to OD_630_ in each well of a 96‐well microplate. SNO‐AGP was added to the culture supernatant, and the cells were statically cultured at 37°C for 9, 24, or 48 hours. After culturing, the medium was stained with 200 µL of 2% (w/v) crystal violet aqueous solution for 30 minutes. Immediately after washing, 200 µL of 95% ethanol was added and decolorized by allowing to stand at 25°C for 30 minutes. Ethanol (100 µL) in the supernatant was transferred to another 96‐well microplate and the biofilm was determined by measuring the absorbance at 570 nm. Data are expressed as mean ± SD (n = 3‐20). ***P < 0.001, **P < 0.01, *P < 0.05, compared with SNO‐AGP (‐)

Biofilm formation is one of the mechanisms of multidrug resistance of bacteria. Biofilm refers to a three‐dimensional structure composed of a microbial population attached to the surface and extracellular polymeric substance (EPS) produced by microorganisms, and microbial cells that formed the biofilm behave differently from cells in a floating state.^26^ It has been reported that various bacteria can form a biofilm and it has also been shown that NO inhibits biofilm formations.^27^ Therefore, to further clarify the mechanism of SNO‐AGP overcoming multidrug resistance, the effect of SNO‐AGP on biofilm formation of *K. pneumoniae* was evaluated by crystal violet method. Figure 4 showed that SNO‐AGP inhibited the biofilm formation of *K. pneumoniae* for at least 48 hours in a concentration‐dependent manner (10^−8^‐10^−7^ M). Such effect was not observed by incubation with AGP. These results suggest that NO released from SNO‐AGP could strongly inhibit biofilm formation through the antimicrobial activity of SNO‐AGP.

### 3.5 Effect of SNO‐AGP on substrate accumulation of multidrug efflux pump

**Figure 5:**
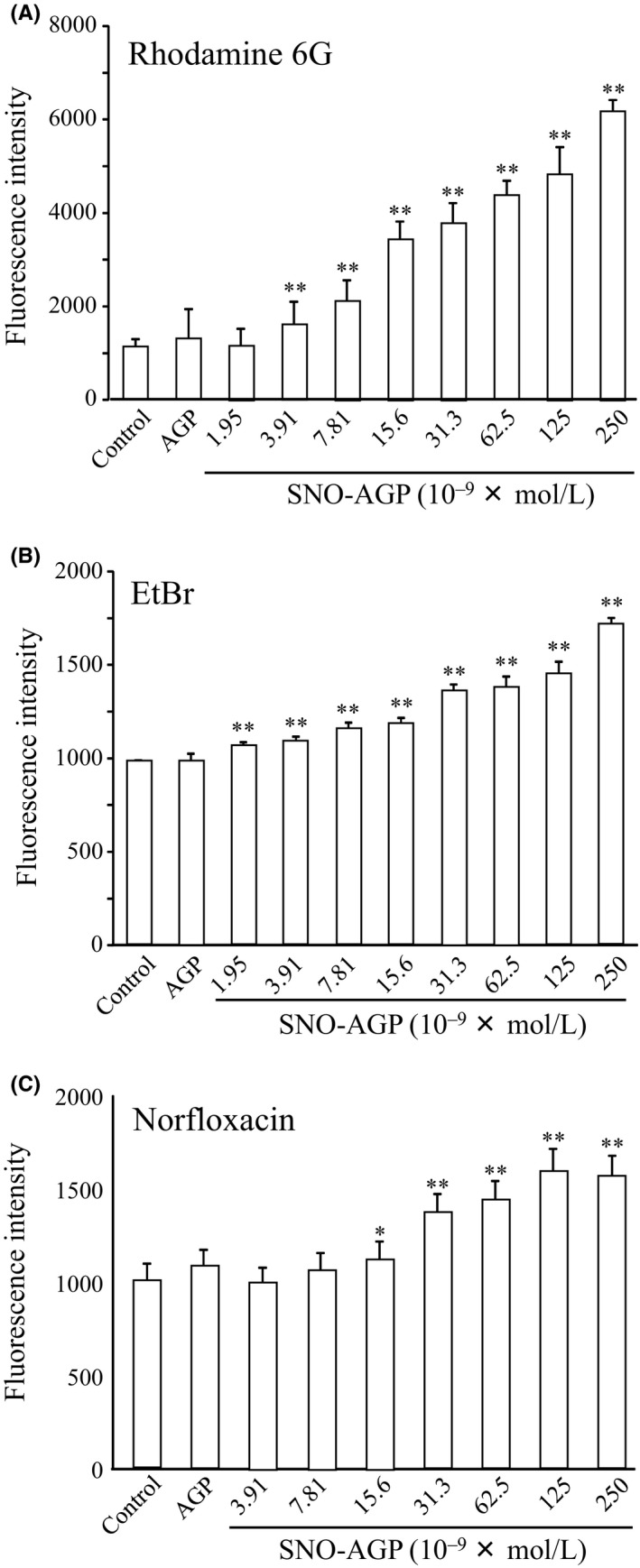
Substrate accumulation of multidrug efflux pump of *Klebsiella pneumoniae* MGH78578 incubated with SNO‐AGP. A multidrug‐resistant strain of *K. pneumoniae* MGH 78578 was grown in M9 medium. Each concentration of SNO‐AGP or AGP (250 nM) was added to the medium supernatant and grown in M9 medium at 37°C. for 5 hours (OD_630_ = 0.2‐0.3). Then, (A) Rhodamine 6G, (B) EtBr, and (C) Norfloxacin as substrates of multidrug efflux pump were added, incubated at 25°C for 15 minutes, and then wash three times in the medium. The measurement was performed at an excitation wavelength of 485 nm and a fluorescence wavelength of 535 nm. Data are expressed as mean ± SD (n = 12). **P < 0.01, *P < 0.05, compared with control

Multidrug efflux pumps were reported as resistance mechanism of *K. pneumoniae* MGH 78578.^22^, ^28^, ^29^, ^30^, ^31^ Hence, we hypothesized that SNO‐AGP synergistically enhances the activity of a broad spectrum of antibacterial agents by inhibiting this multidrug efflux pump. In order to examine this hypothesis, we measured bacterial accumulation of Rhodamine 6G, ethidium bromide (EtBr), and norfloxacin, which are substrates of a multidrug efflux pump, in the presence of SNO‐AGP. A significant increase in the cellular fluorescence intensities was observed in a depending on the concentration of SNO‐AGP dependent manner. Moreover, no increase in fluorescence intensity was observed when AGP was added at the same concentration (Figure 5). These indicated that SNO‐AGP could overcome the *K. pneumoniae* MGH 78578 resistance via multidrug efflux pumps inhibition. Furthermore, the reaction time with SNO‐AGP was varied from 0 to 9 hours, and the accumulation of Rhodamine 6G was evaluated. At this time, SNO‐AGP was added at a concentration of 0.015 μM, which does not exhibit the growth‐suppressing effect (Supporting information Figure S1). As a result, the elevating effect of Rhodamine 6G accumulation by SNO‐AGP was observed from 3 hours after incubation and lasted until 6 hours, but it disappeared after 9 hours thereafter (Supporting information Figure S2), suggesting that effect of SNO‐AGP on the activity of multidrug efflux pumps is transiently inhibited.

### 3.6 Effect of SNO‐AGP on AcrAB‐TolC efflux activity of multidrug efflux pump

**Figure 6:**
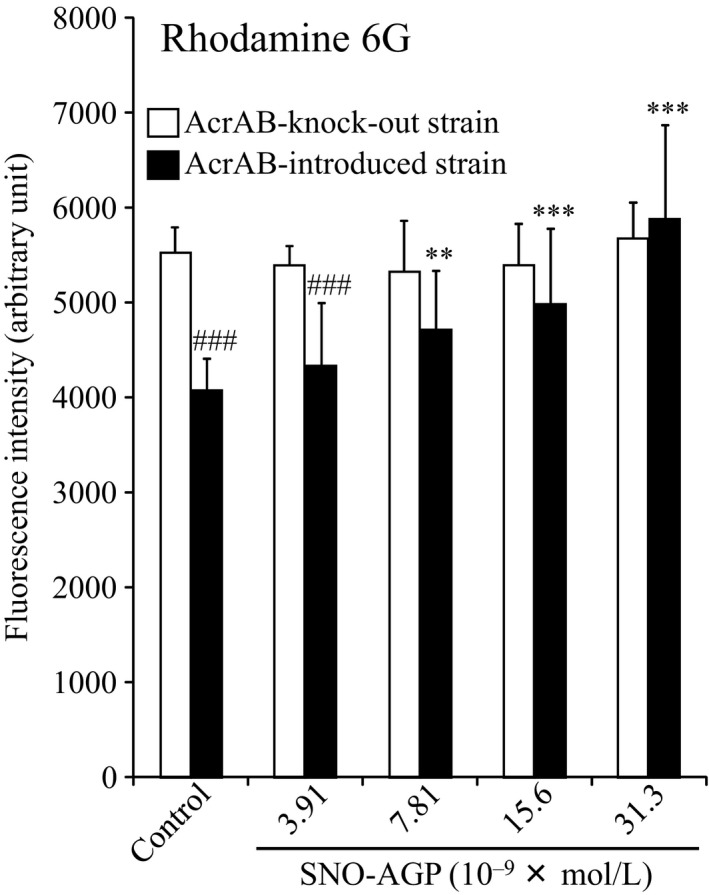
Efflux activities of Rhodamine 6G in AcrAB‐knockout stain of *Klebsiella pneumonia*. AcrAB‐knockout strain (SKY2/pSTV28, *acrAB*‐disrupted strain from *K. pneumoniae* ATCC10031) and AcrAB‐introduced strain (SKY2/pKAC28M, *acrAB*‐transformed strain for SKY2/pSTV28) were grown in M9 medium. Each concentration of SNO‐AGP was added to the medium supernatant and grown in M9 medium at 37°C for 5 hours. Then, Rhodamine 6G was added, incubated at 25°C for 15 minutes, and then washed three times in the medium. The measurement was performed at an excitation wavelength of 485 nm and a fluorescence wavelength of 535 nm. Data are expressed as mean ± SD (n = 8). ***P < 0.001, **P < 0.01, compared with AcrAB‐introduced stain, SNO‐AGP (‐). ###*P *< 0.001, compared with AcrAB‐knockout stain, SNO‐AGP (‐)

*K. pneumonia* MGH78578 expresses multidrug efflux pump, AcrAB‐TolC belonging to RND family, its multidrug resistance is known to involve this efflux pump.^30^ To clarify the effects of SNO‐AGP on AcrAB‐TolC, we investigated the accumulation of Rhodamine 6G using AcrAB‐deficient strain (AcrAB‐knockout stain from *K. pneumoniae* ATCC10031) compared with AcrAB‐introduced strain. As a result, SNO‐AGP could increase the Rhodamine 6G fluorescence of AcrAB‐introduced stain in a concentration‐dependent manner. On the other hand, the Rhodamine 6G fluorescence of AcrAB‐knockout stain was not affected by SNO‐AGP treatment (Figure 6). This data indicated that SNO‐AGP increases the accumulation of substrate by suppressing multidrug efflux pump AcrAB‐TolC.

### 3.7 Effect of SNO‐AGPs on bacterial ATP levels

**Figure 7:**
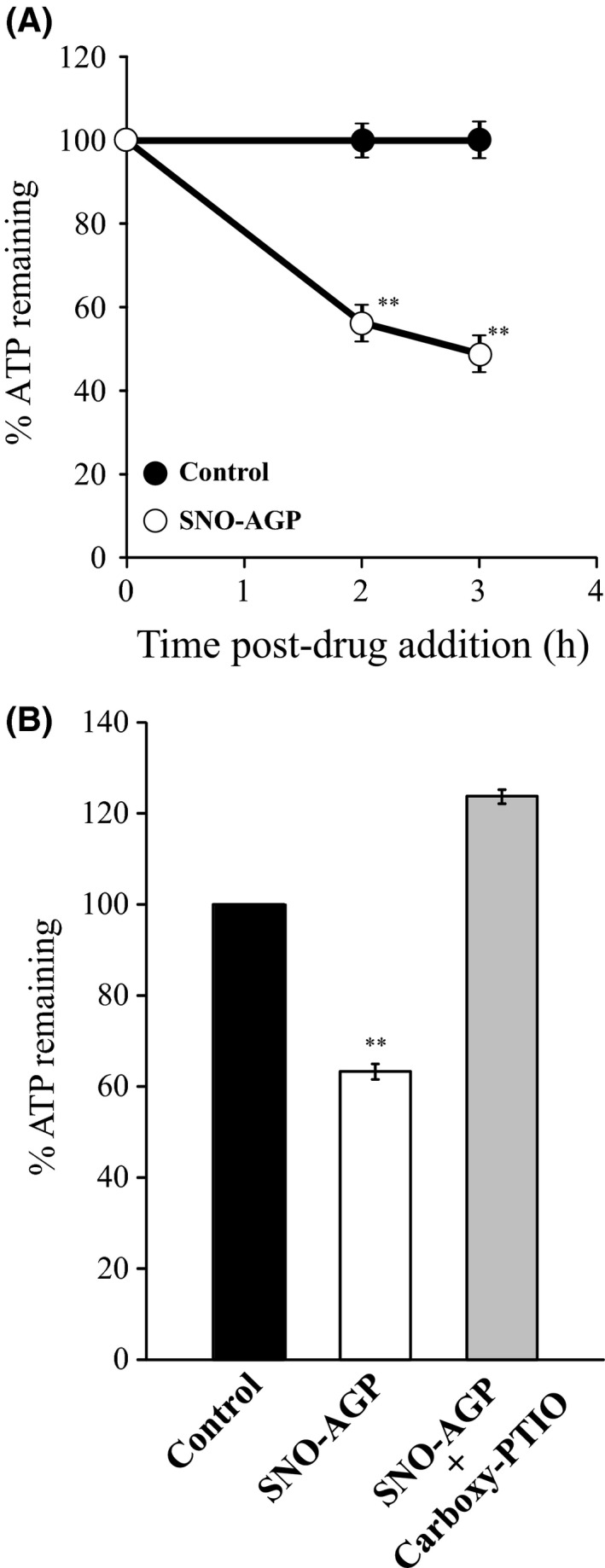
ATP levels on bacteria after incubation of SNO‐AGP. After reacting *Klebsiella pneumoniae* with 250 nM SNO‐AGP at 37°C, an equal amount of reagent was added to the medium supernatant and incubated at 25°C for 5 min, then ATP level on bacteria was measured using the BacTiter‐Glo ™ Microbial Cell Viability Assay. Effect of 100 pM Carboxy‐PTIO on SNO‐AGP reduction of ATP level in bacteria was also performed. Data are expressed as means ±SD (n = 3). **P < 0.01, compared with control

We previously demonstrated that SNO‐AGP possesses a very strong antibacterial activity against various bacteria via NO transfer to bacteria.^14^ It is well‐known that NO inhibits cytochrome c oxidase in the electron transport chain, thereby decreasing the ATP level in bacteria.^31^ Therefore, the influence of SNO‐AGP on ATP level in bacteria was evaluated. The results showed that ATP level in bacteria was decreased by SNO‐AGP within 2 hours of addition, and it was reduced to about 50% after 3 hours (Figure 7A). The inhibitory effect of SNO‐AGP was canceled by carboxy‐PTIO which is a NO scavenger (Figure 7B). These results suggest that SNO‐AGP could reduce the bacterial ATP level in a NO‐dependent manner.

### 3.8 Generation of active oxygen species (ROS) by SNO‐AGP

**Figure 8:**
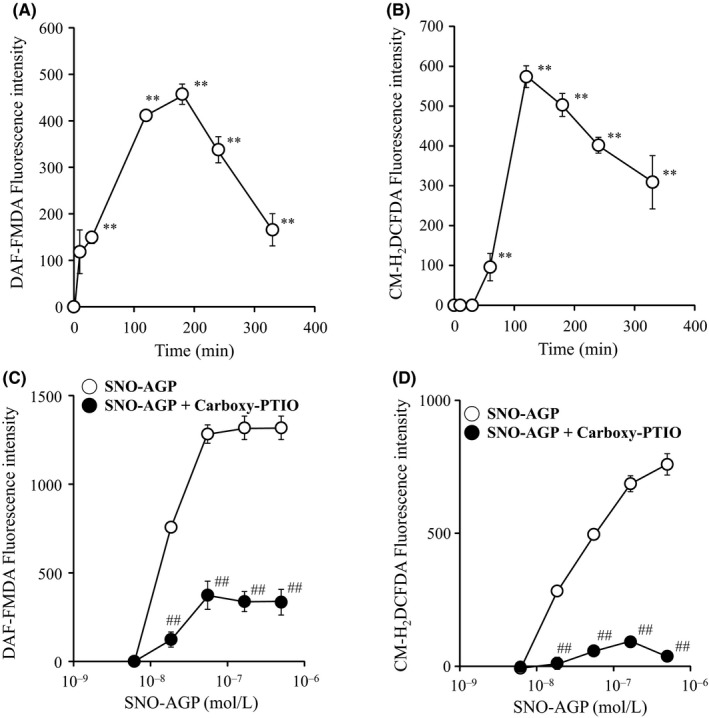
Intraceller NO and ROS production in bacteria stimulated by SNO‐AGP. *Klebsiella pneumoniae* was prepared in M9 medium, and each SNO‐AGP was added to the medium supernatant and reacted at 37°C for 7 hours. Thereafter, DAF‐FMDA (for NO) or CM‐H_2_DCFDA (for ROS) was added and reacted at 37°C for 1 hour. SPECTRA FLUOR XFluor 4 (TECAN) was used for monitoring at an excitation wavelength of 485 nm and a fluorescence wavelength of 535 nm. Effect of Carboxy‐PTIO on the intracellular NO and ROS production stimulated by SNO‐AGP was also performed. Data are expressed as means ± SD (n = 9). **P < 0.01, compared with SNO‐AGP (‐). ##*P *< 0.01, compared with SNO‐AGP alone

**Figure 9:**
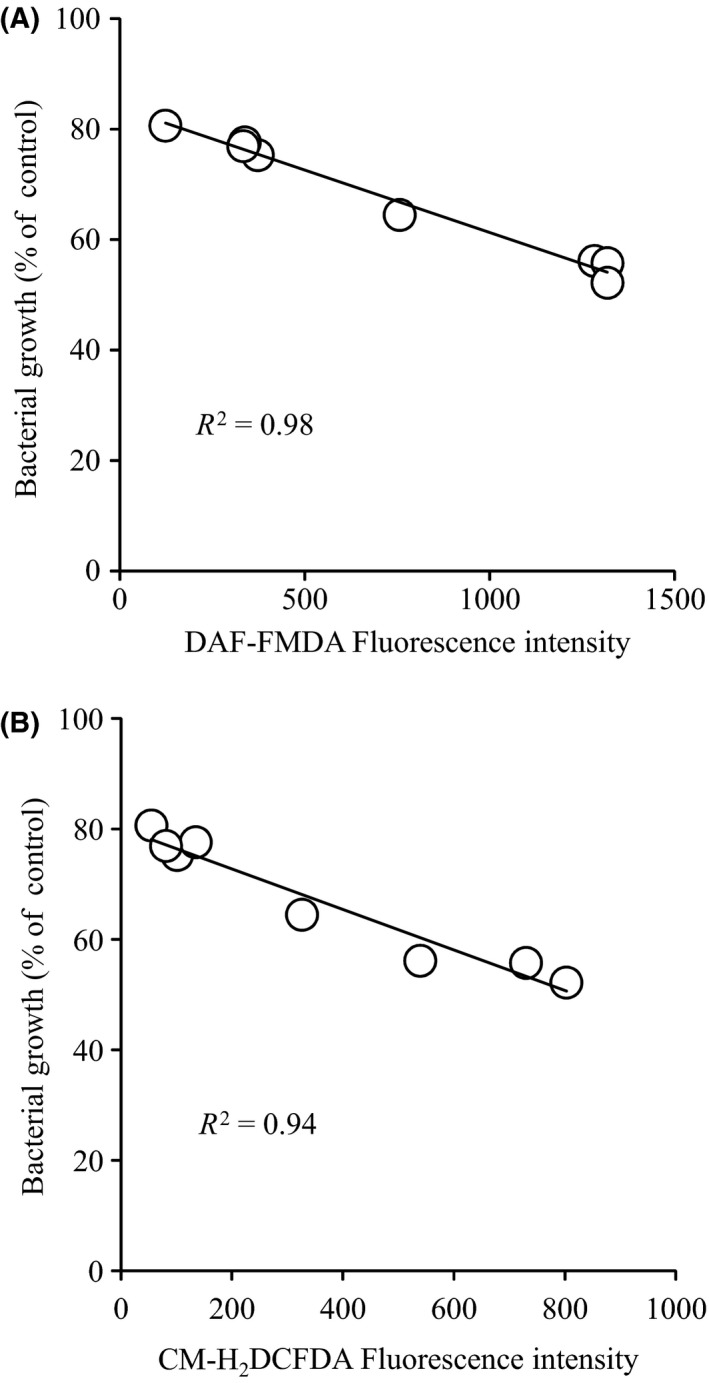
Correlation of (A) NO or (B) ROS levels and bacterial growth. Linear regression of logarithmic values was calculated by using the least squares method (A) r = 0.98, (B) r* = *0.94

The possibility of reactive oxygen species (ROS) generation by SNO‐AGP via cytochrome c oxidase inhibition was analyz ed using the ROS detection fluorescent probe CM‐H_2_DCFDA.^32^ DAF‐FMDA fluorescence (NO probe) increased within 10 minutes, whereas fluorescence intensity of CM‐H_2_DCFDA increased 30 minutes later (Figure 8AB) after SNO‐AGP addition. ROS generation in bacteria was also inhibited by carboxy‐PTIO (Figure 8C and D). Furthermore, the bacterial growth was negatively correlated with both DAF‐FMDA and CM‐H_2_DCFDA (Figure 9). These results suggest that one of the antibacterial mechanisms of SNO‐AGP was derived from ATP depletion and ROS generation induced by the inhibition of cytochrome c oxidase.

### 3.9 Nitrotyrosine induced by SNO‐AGP in Bacteria

**Figure 10:**
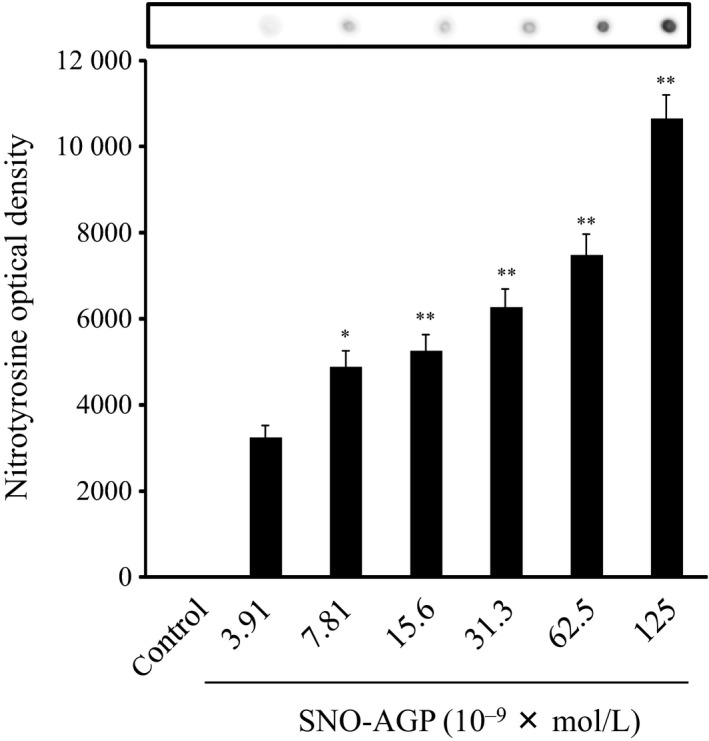
Nitrotyrosin formation in bacteria induced by SNO‐AGP *Klebsiella pneumoniae* was reacted with SNO‐AGP at 37°C for 30 minutes. The SNO‐AGP‐treated bacteria was lysed, transferred to a nitrocellulose membrane, and the membrane was dried for 15 minutes at 60°C. For detection of nitrated protein, a mouse monoclonal 3‐nitrotyrosine antibody was used. Densitometric quantification of the dots was performed using ImageJ software. Data are means of triplicate experiments ±SD. *P < 0.05, **P < 0.01, compared with SNO‐AGP (‐)

The presence of ROS and NO could induce peroxynitrite (ONOO^−^) in bacteria. The generation of ONOO^−^ can be evaluated by detecting the nitration of endogenous proteins in bacteria using dot‐blotting analysis. Figure 10 showed that SNO‐AGP could induce nitration of whole bacteria proteins in a concentration‐dependent manner. This suggests that the function of some proteins such as AcrAB‐TolC might be inhibited via nitration of active sites.

## 4 DISCUSSION

We previously reported that SNO‐AGP kills drug‐resistant bacteria and aids survival in sepsis. Subsequently, we demonstrated here two major findings: (a) SNO‐AGP synergistically enhances the activity of a wide range of antimicrobial agents, and (b) SNO‐AGP could act as an antibacterial agent and chemical sensitizer via inhibiting biofilm formation, multidrug efflux pump and ATP levels in bacteria. These findings indicated that the sensitivity of antimicrobial agents that have become ineffective due to multidrug resistance could be revived when use in combination with SNO‐AGP. However, further in vivo studies are needed to examine whether sufficient clinical effect can be obtained in actual clinical application.

SNO‐AGP enhanced the antimicrobial activity of ineffective antibiotics against *K. pneumoniae* via mechanisms involving inhibitions of biofilm formation (Figure 4) and multidrug efflux pump (Figures 5 and 6). Biofilm formation inhibition by SNO‐AGP is expected to increase the amount of antibiotic reaching the bacteria.^33^, ^34^, ^35^ Active efflux of antibiotics by multidrug efflux pumps is also an important mechanism in multidrug resistance of bacteria.^36^ Among the multidrug efflux pumps, the multidrug efflux systems similar to AcrAB are expressed not only in *K. pneumoniae*, but also in a wide range of gram‐negative multidrug resistance bacteria including *E. coli*.^37^, ^38^, ^39^ AcrAB is composed of the inner membrane protein AcrB belonging to the RND family^40^ and the periplasmic lipoprotein AcrA belonging to the membrane fusion protein family.^41^ In cooperation with the outer‐membrane channel TolC,^42^ the AcrAB‐TolC efflux pump is able to transport vectorially a diverse array of compounds with little chemical similarity, thus conferring resistance to a broad spectrum of antibiotics using the proton (H^+^) driving force.^43^, ^44^ The research findings on the crystal structure analysis^45^, ^46^, ^47^ and expression regulation^48^, ^49^, ^50^ illuminate the basis for drug resistance in numerous pathogenic bacterial species. However, clinically applicable inhibitor of multidrug efflux pumps like AcrAB is still yet to be discovered. Hence, SNO‐AGP has high potential as the first multidrug efflux pump inhibitor to overcome the multidrug resistance of a wide range of gram‐negative bacteria expressing AcrAB‐TolC. Therefore, further human studies are needed to examine whether inhibitor of multidrug efflux pumps like AcrAB can be affected on the effect of other antibiotics against multidrug resistance bacterial infection. In addition, Tables 2 and 3 showed that antibacterial activity of some antibiotics such as kanamycin could be reduced by SNO‐AGP. The inhibitory mechanism of some antibiotics such as kanamycin by SNO‐AGP should be clarified in near future.

The antibiotics whose activity was enhanced by SNO‐AGP against *K. pneumoniae*, oxacillin, norfloxacin, erythromycin, and tetracycline have been shown to be a substrate of AcrAB‐TolC.^20^ In contrast, cefmetazole, kanamycin, and chloramphenicol that are not considered to be a substrate of AcrAB‐TolC, did not exhibit the synergistic effect. Although imipenem is not a substrate of AcrAB‐TolC, an enhanced activity by SNO‐AGP combination was observed. Porin pore expressed on the outer membrane of bacteria involved as a major resistance mechanism against imipenem. Previous reports showed that low expression of porin pore inhibits the uptake of imipenem into the bacteria.^51^, ^52^ It is possible that SNO‐AGP affects the expression of porin pore, thereby increasing the uptake of imipenem and enhancing the activity, but detailed investigation is needed in the future.

Emergence of various multidrug‐resistant bacteria causing resistant bacterial infection is a very critical clinical problem. SNO‐AGP was induced in bacterial‐infected mice, indicated that SNO‐AGP is one of the host defense mechanism. To clarify whether SNO‐AGP acts as host defense factor in human, detection of SNO‐AGP in human plasma during various bacterial infection should be further investigated. In conclusion, SNO‐AGP, a novel inhibitor of multidrug efflux pumps, is highly promising as a new drug for designing a new therapeutic strategy to overcome multidrug resistance bacterial infection conundrum.

## CONFLICT OF INTEREST

Authors report no disclosures.

## AUTHOR CONTRIBUTIONS

Y. Ishima, M. Otagiri and T. Maruyama designed research; K. Watanabe and I. Takeda performed most of research; T. Kuroda and W. Ogawa contributed the experiments using multidrug‐resistant strains; Y. Ishima, V. T. G. Chuang, H. Watanabe, Y. Iwao, and T. Ishida analyzed data; Y. Ishima and V. T. G. Chuang wrote the paper; and all authors reviewed the manuscript.
