# Supplementary figures and images for: S‐Nitrosated alpha‐1‐acid glycoprotein exhibits antibacterial activity against multidrug‐resistant bacteria strains and synergistically enhances the effect of antibiotics
Source: FASEB Bioadv. 2019 Feb 4;1(3):137–50. doi: 10.1096/fba.1018 (PMC6996401; doi:10.1096/fba.1018)

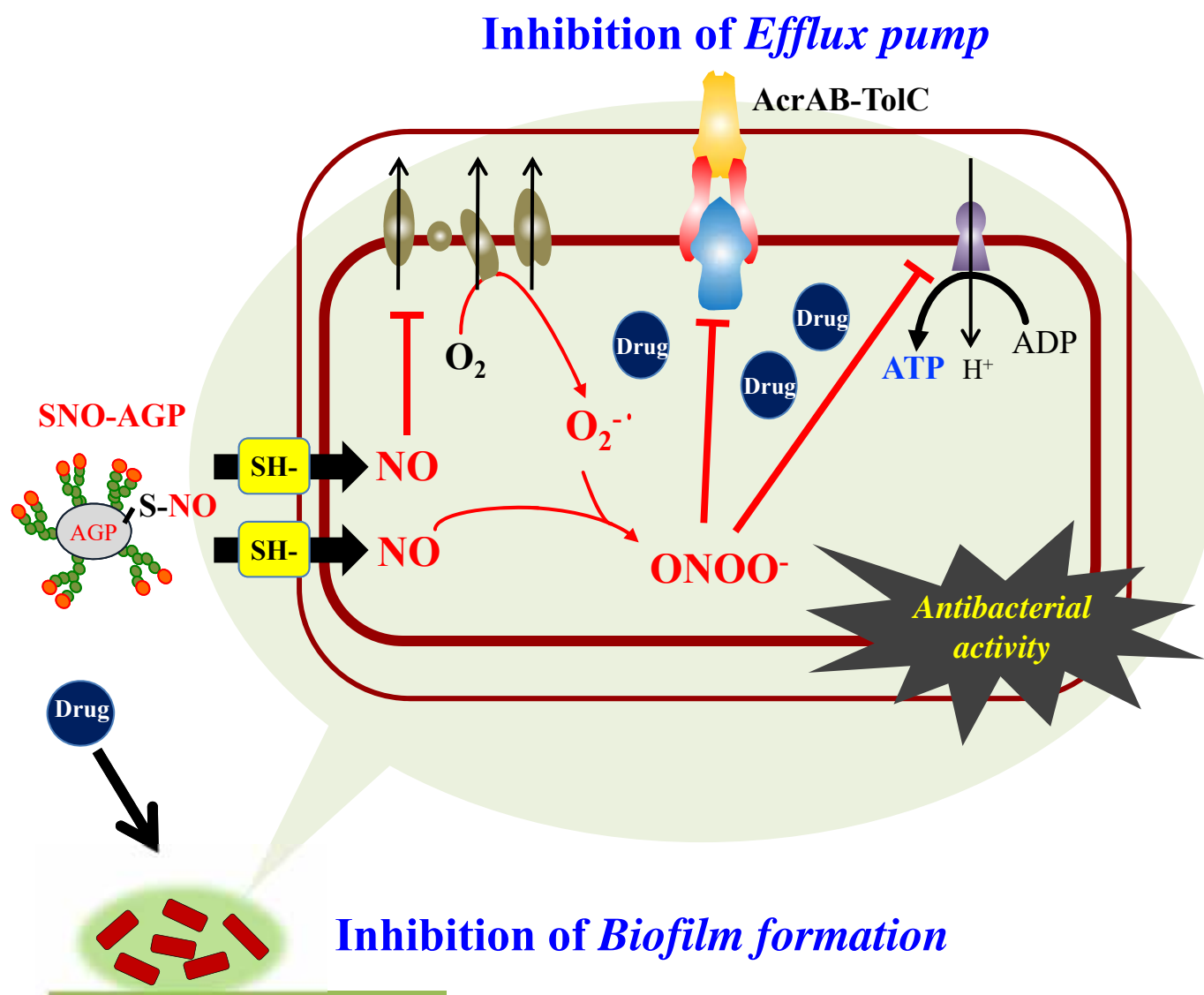

Supplement: Supplementary file 1 [file FBA2-1-137-s001.pdf]
