# Supplementary material for: S‐Nitrosated alpha‐1‐acid glycoprotein exhibits antibacterial activity against multidrug‐resistant bacteria strains and synergistically enhances the effect of antibiotics
Source: FASEB Bioadv. 2019 Feb 4;1(3):137–50. doi: 10.1096/fba.1018 (PMC6996401; doi:10.1096/fba.1018)
Supplement: Supplementary file 2 [file FBA2-1-137-s002.pdf]

Supplemental Figure 1

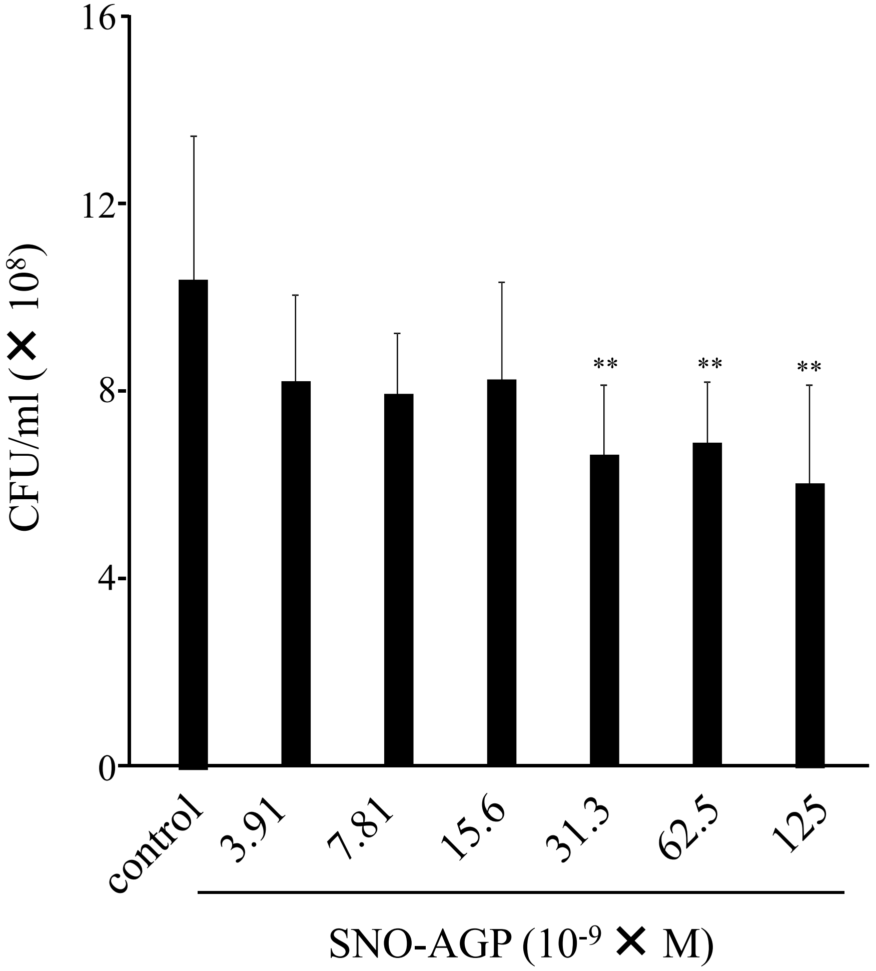

Supplemental Figure 1 Colony Forming Unit of *K. pneumoniae* MGH78578 treated with SNO-AGP
Data are expressed as mean ± S.D. (n= 9). **$p < 0.01$, compared with SNO-AGP (-).

Supplemental Figure 2

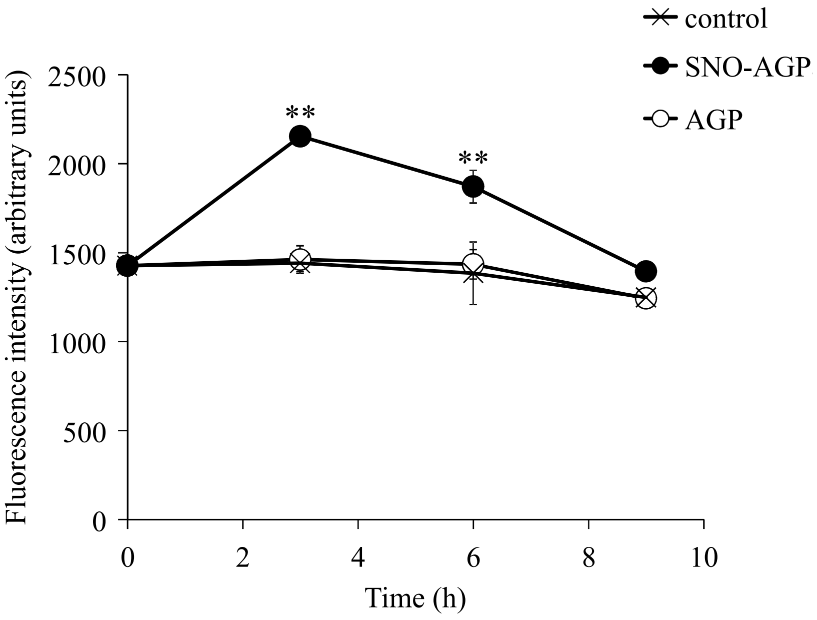

Supplemental Figure 2 Rhodamine 6G accumulation in *K. pneumoniae* MGH78578 incubated with SNO-AGP for indicated periods

Data are expressed as mean ± S.D (n= 6). ** $p < 0.01$, compared with control.
